# Deficient Chaperone-Mediated Autophagy Promotes Lipid Accumulation in Macrophage

**DOI:** 10.1007/s12265-020-09986-3

**Published:** 2020-04-13

**Authors:** Lei Qiao, He-feng Wang, Lei Xiang, Jing Ma, Qiang Zhu, Dan Xu, Hui Zheng, Jie-qiong Peng, Sen Zhang, Hui-xia Lu, Wen-qiang Chen, Yun Zhang

**Affiliations:** 1 The Key Laboratory of Cardiovascular Remodeling and Function Research, Chinese Ministry of Education, Chinese National Health Commission and Chinese Academy of Medical Sciences, The State and Shandong Province Joint Key Laboratory of Translational Cardiovascular Medicine, Department of Cardiology, Qilu Hospital of Shandong University, 107 Wenhuaxi Road, 250012 Jinan, China; 2 Qilu Hospital of Shandong University (Qingdao), No. 758 Hefei Road, Qingdao, 266035 China; 3 Department of Cardiology, Sishui County People’s Hospital, Sishui, 273200 Shandong China; 4 Department of clinical laboratory, Sishui County People’s Hospital, Sishui, 273200 Shandong China; 5 School of Medicine and Life Sciences, University of Jinan-Shandong Academy of Medical Sciences, Jinan, China

**Keywords:** Atherosclerosis, Chaperone-mediated autophagy, Lipid metabolism, Long-chain-fatty-acid-CoA ligase 1, Lysosomal acid lipase

## Abstract

Chaperone-mediated autophagy (CMA) serves as a critical upstream regulator of lipophagy and lipid metabolism in hepatocyte. However, the role of CMA in lipid metabolism of macrophage, the typical component of atherosclerotic plaque, remains unclear. In our study, LAMP-2A (L2A, a CMA marker) was reduced in macrophages exposed to high dose of oleate, and lipophagy was impaired in advanced atherosclerosis in ApoE (−/−) mice. Primary peritoneal macrophages isolated from macrophage-specific L2A-deficient mice exhibited pronounced intracellular lipid accumulation. Lipid regulatory enzymes, including long-chain-fatty-acid-CoA ligase 1 (ACSL1) and lysosomal acid lipase (LAL), were increased and reduced in L2A-KO macrophage, respectively. Other lipid-related proteins, such as SR-A, SR-B (CD36), ABCA1, or PLIN2, were not associated with increased lipid content in L2A-KO macrophage. In conclusion, deficient CMA promotes lipid accumulation in macrophage probably by regulating enzymes involved in lipid metabolism. CMA may represent a novel therapeutic target to alleviate atherosclerosis by promoting lipid metabolism.

**Electronic supplementary material:**

The online version of this article (10.1007/s12265-020-09986-3) contains supplementary material, which is available to authorized users.

**Figure:**
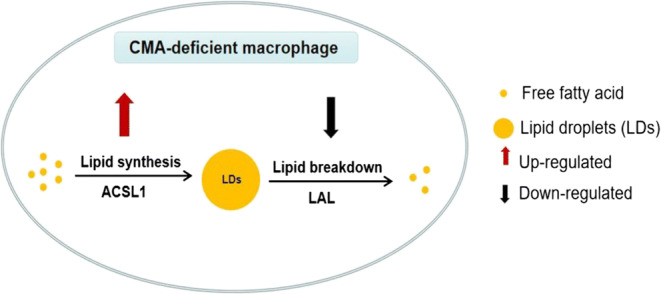
Graphical abstract

## Introduction

Macrophages, the main cellular population in atherosclerotic plaque, play vital roles in the development of atherosclerosis [1]. Macrophages uptake modified low-density lipoprotein (LDL) and transform into foam cells, which marks the formation of atherosclerotic lesion [1]. The internalized lipids are stored in the form of lipid droplet (LD). Therefore, how to reduce lipid accumulation in macrophage foam cells is a potential therapeutic target for the treatment of atherosclerosis and has always attracted the attention of scientists.

In the past decade, an encouraging finding is the contribution of autophagy to lipid catabolism. First reported in 2009 by Rajat Singh [2], autophagy can regulate intracellular lipid stores. Subsequent study illustrated that autophagy regulates cholesterol efflux from macrophage foam cells via lysosomal acid lipase (LAL) [3]. These studies spawn the word “lipophagy,” which refers to LD breakdown through autophagy-lysosome pathway [4]. However, studies relating to autophagy and lipid metabolism are somehow incomplete because there are three types of autophagy: macroautophagy, microautophagy, and CMA. Most of the emphasis has been put on macroautophagy (often called autophagy), while its cousin, CMA, is less well studied. CMA is a selective form of autophagy that participates in the lysosomal degradation of special cytosolic proteins [5]. All substrates for CMA are recognized and bound by HSC70 (heat shock cognate 71 kDa protein) and transported to the lysosomal surface, where the substrate protein/HSC70 complex binds the lysosome-associated membrane glycoprotein 2 isoform A (LAMP-2A) [5]. LAMP-2A serves as a receptor for CMA via its cytoplasmic tail and, in fact, is exclusive for CMA. CMA was thought to play a role only in protein degradation, but it was also found to promote glucose and lipid metabolism and overall organism energetics. Specifically, Susmita Kaushik [6] reported that CMA facilitated lipolysis by degradation of lipid droplet-associated proteins and deficient CMA in mouse liver caused lipid and carbohydrate metabolic abnormalities [7], positioning CMA as a critical upstream regulator of both macrolipophagy and cytosolic lipolysis. These elegant studies shed new light on human diseases with lipid over-accumulation syndrome.

These findings prompt us to further investigate the role of CMA in regulating macrophage lipid homeostasis, which may have important implications for the basis of atherosclerosis. To reach this goal, we generated a macrophage-specific conditional knockout mouse for LAMP-2A, the most important component of CMA, to selectively block CMA in macrophage and study the physiological function of CMA in macrophage lipid metabolism.

## Method

### Animals

Twenty C57BL/6 mice and twenty ApoE (−/−) mice (male, 8 weeks old) were obtained from the Charles River Laboratories (Beijing, China). The mice were given different time of high fat diet (HFD) to generate two stages of atherosclerosis (“early” versus “advanced” lesions as defined by the duration of HFD, 8 weeks and 20 weeks, respectively) [8]. All animal experimental protocols were approved by the Ethical Committee of Qilu Hospital of Shandong University.

Macrophage-specific LAMP-2A (L2A)-deficient mice in a C57BL/6 background were generated by crossing L2A floxed mice (L2A^fl/fl^ mice, Viewsolid Biotech, Beijing, China) with LysM-cre mice (Jackson Laboratory). L2A^fl/fl^ LysM-cre (L2A-KO) mice were selected as experimental group, and its littermates L2A^fl/fl^ LysM-cre (−) mice were used as control (CTR).

### Macrophage Culture and Treatment

Primary peritoneal macrophages were obtained from L2A-KO mice and control mice as described [9]. Briefly, the mice (8 weeks old) were injected intraperitoneally with 3% sterile starch, 1 ml/mouse. Three days later, peritoneal macrophages were harvested by repeatedly perfusing the peritoneal cavity with cold PBS. Similar numbers of cells were obtained from both genotypes. Cells were centrifugated, resuspended, counted, and plated in DMEM (Gibco) with 10% FBS (Gibco) and 1% antibiotics (Gibco). After 3 h, the fluid was changed to remove the non-adherent cells. To perform foam cell assay, macrophages were cultured on sterile coverslips and treated with sodium oleate (OL; 0.06 mM, Sigma-Aldrich, SLBR5187V) in a serum-free medium for 24 h.

### Macrophage Lipid Accumulation Assay

LDs were stained using Bodipy 493/503 (7 μg/mL, 790,389, Sigma, USA) or Oil-red-O (Yiyuan biotechnology, Guangzhou, China) for 30 min prior to visualization.

### Immunofluorescence

Mice were sacrificed with pentobarbital, and the heart with attached aortic roots was obtained immediately, fixed in 4% paraformaldehyde overnight, embedded in optimum cutting temperature (OCT), and then cut into serial 6-um cross sections for immunofluorescence. Lipid accumulation was stained with Bodipy 493/503 (7 μg/mL, 790389, Sigma, USA). Rabbit anti-LC3B antibody (ab48394, Abcam, UK) was used to analyze LC3B staining.

### Western Blotting

Proteins obtained from cells were isolated, and equal amounts of protein (25 μg) were separated by 10% or 12% SDS-PAGE gel electrophoresis. Proteins were transferred to PVDF membranes (0.22 μm, Bio-Rad, USA) after electrophoresis. The membranes were blocked with 5% milk for at least 1 h and incubated with primary antibodies: rabbit polyclonal to LC3B (ab48394, Abcam, UK), rabbit monoclonal to Atg5 (ab108327, Abcam, UK), LAMP-2A (AMC2, Invitrogen, USA), rabbit monoclonal to SR-A (ab151707, Abcam, UK), rabbit monoclonal to CD36 (ab133625, Abcam, UK), rabbit polyclonal to LAL (ab154356, Abcam, UK), guinea pig polyclonal to perilipin 2 (GP40, Progen Biotechnik), and rabbit polyclonal to beta actin (ab8227, Abcam, UK).

### Statistical Analysis

All experiments were repeated at least 3 times. All numerical results are reported as mean ± standard error of the mean. Categorical data were expressed as number (%). Comparison of continuous variables between two groups was analyzed by Student’s *t* test for normally distributed variables. The chi-squared test was used for comparing categorical data. All analyses were performed with SPSS 16.0 (SPSS Inc., Chicago, IL, USA). *p* < 0.05 was considered statistically significant.

## Results

### Progressive Atherosclerosis Has Features of Dysfunctional Lipophagy

**Fig. 1:**
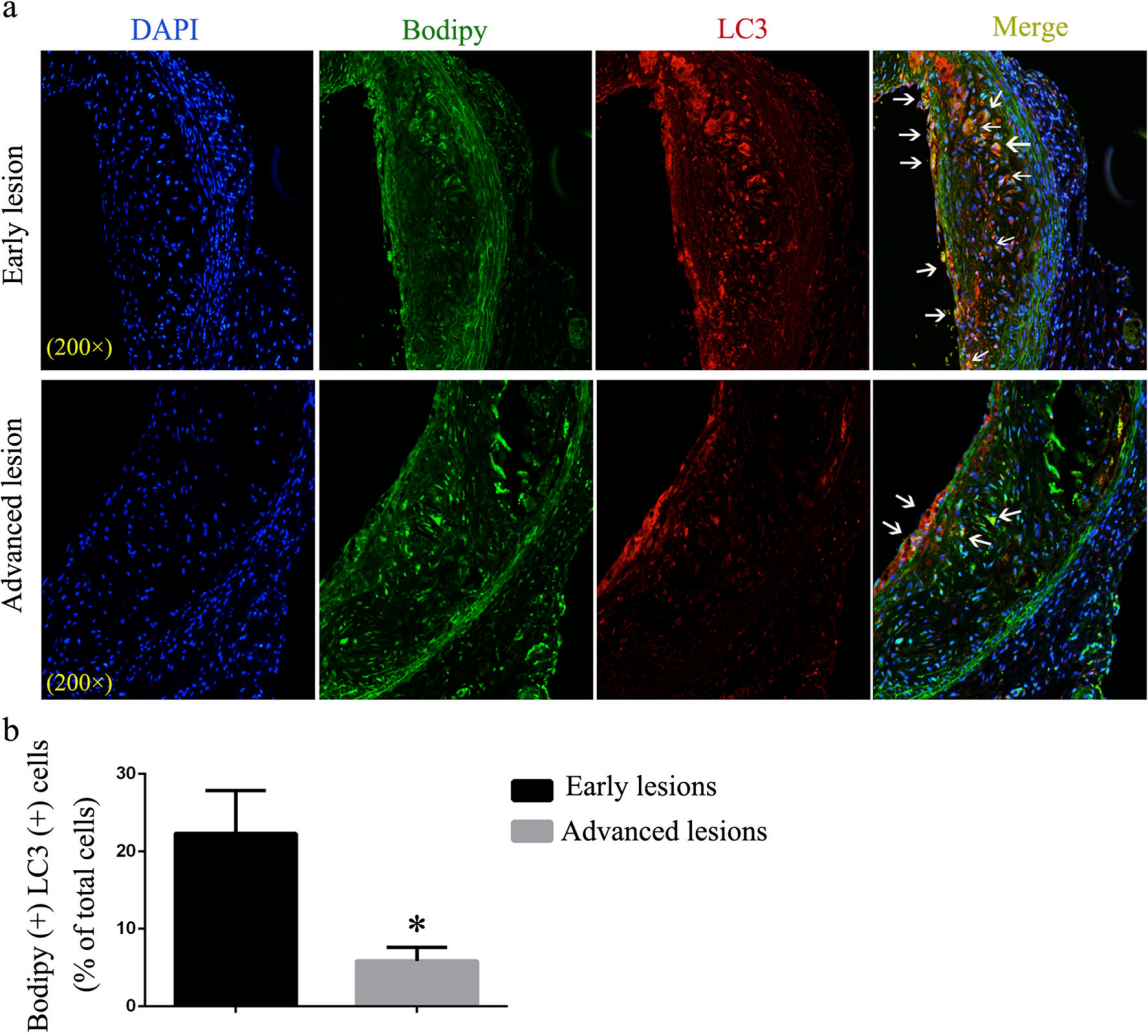
Immunofluorescence analysis of lipophagy in “early atherosclerotic lesions” and “advanced atherosclerotic lesions.” **a** LDs were stained using Bodipy 493/503. Co-localization of LC3 and LDs represents the interaction between autophagosome and LDs. Arrowheads indicate Bodipy^+^ LC3^+^ double-positive areas (white). **b** Statistics of the percent of Bodipy^+^ LC3^+^ cells in atherosclerotic lesions of the two groups. *p < 0.05; *n* = 3

Lipophagy refers to lipolysis of LDs through macroautophagy-lysosomal pathway, which is reported to be regulated by CMA [5]. Co-localization of LDs (stained using Bodipy) and autophagosomal marker LC3 are used as one of the methods to assess lipophagy activity [2, 3, 10]. Therefore, we first performed double immunofluorescence labeling at two stages of atherogenesis (“early” versus “advanced” lesions as defined by the duration of HFD). Early lesions exhibited high levels of LC3 co-localized with LDs, suggesting a direct association between LD surface and autophagosomes (Fig. 1a–b). However, co-localization of LC3 and LDs reduced considerably in advanced lesions (Fig. 1a–b). This result suggests that lipophagy is impaired in advanced atherosclerotic lesions and its dysfunction may be responsible for lipid accumulation and atherosclerosis progression.

### High Doses of Oleate Impairs Both Macroautophagy and CMA in Macrophage

**Fig. 2:**
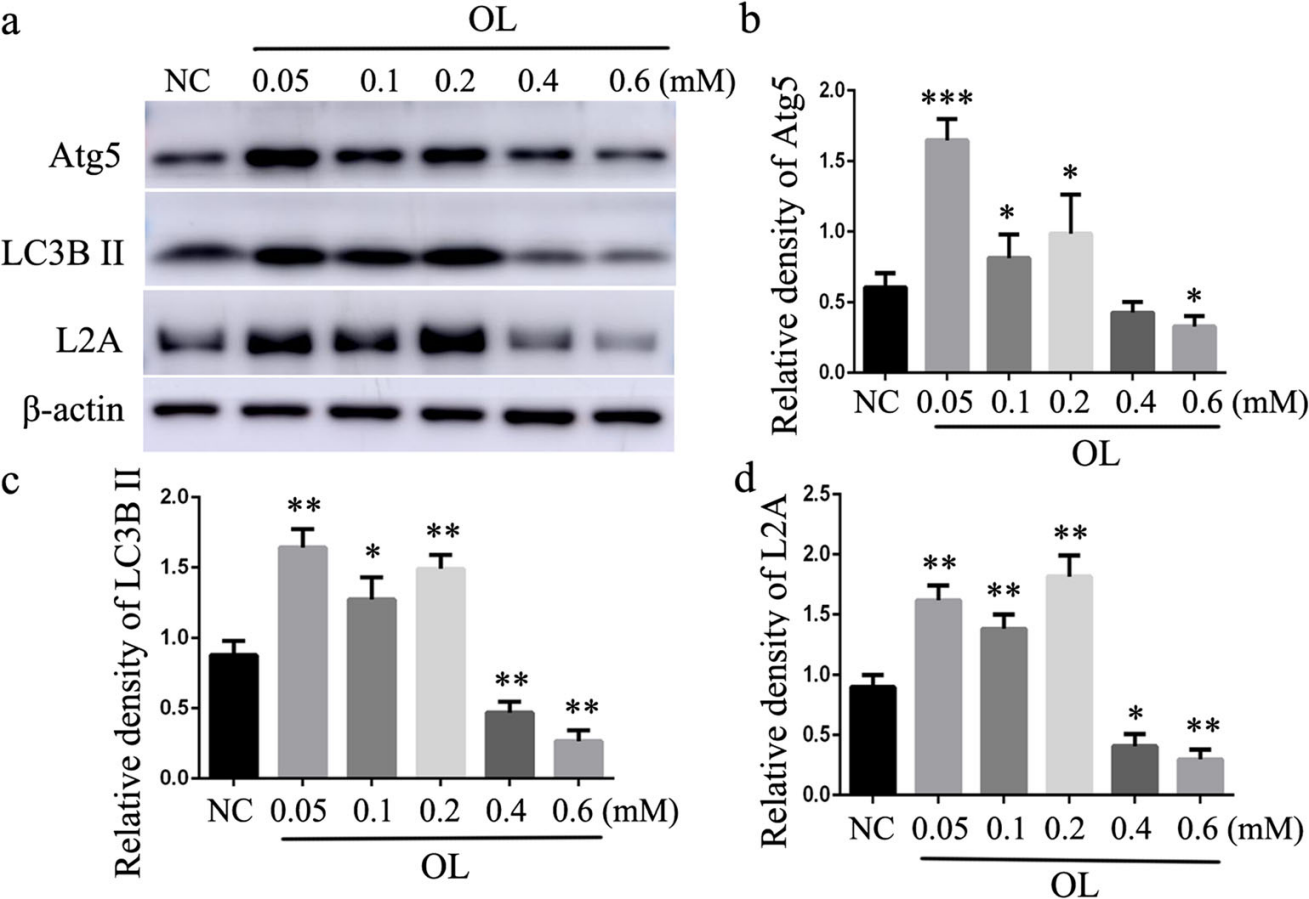
Effect of different doses of OL on macroautophagy and CMA in macrophage. **a–c** Western blot for macroautophagy marker Atg5 and LC3B. **a** and **d** Western blot for CMA marker LAMP-2A (L2A). *p < 0.05, **p < 0.01, and ***p < 0.001 compared with control (NC), *n* = 3

Atherosclerosis progression is accompanied with increasing lipid accumulation in the arterial wall. In order to simulate this pathological process, we treated mouse peritoneal macrophages with different doses of OL for 24 h. The lower concentration of OL upregulated the expression of macroautophagy markers (Atg5 and LC3B) and CMA marker LAMP-2A (Fig. 2). However, high concentration of OL had the opposite effects. These data suggest that both macroautophagy and CMA are impaired in lipid-loaded macrophage. Not only does macroautophagy play an important role in lipid metabolism as previously reported but CMA may be equally important.

### Deficient CMA Promotes Lipid Accumulation in Macrophage

**Fig. 3:**
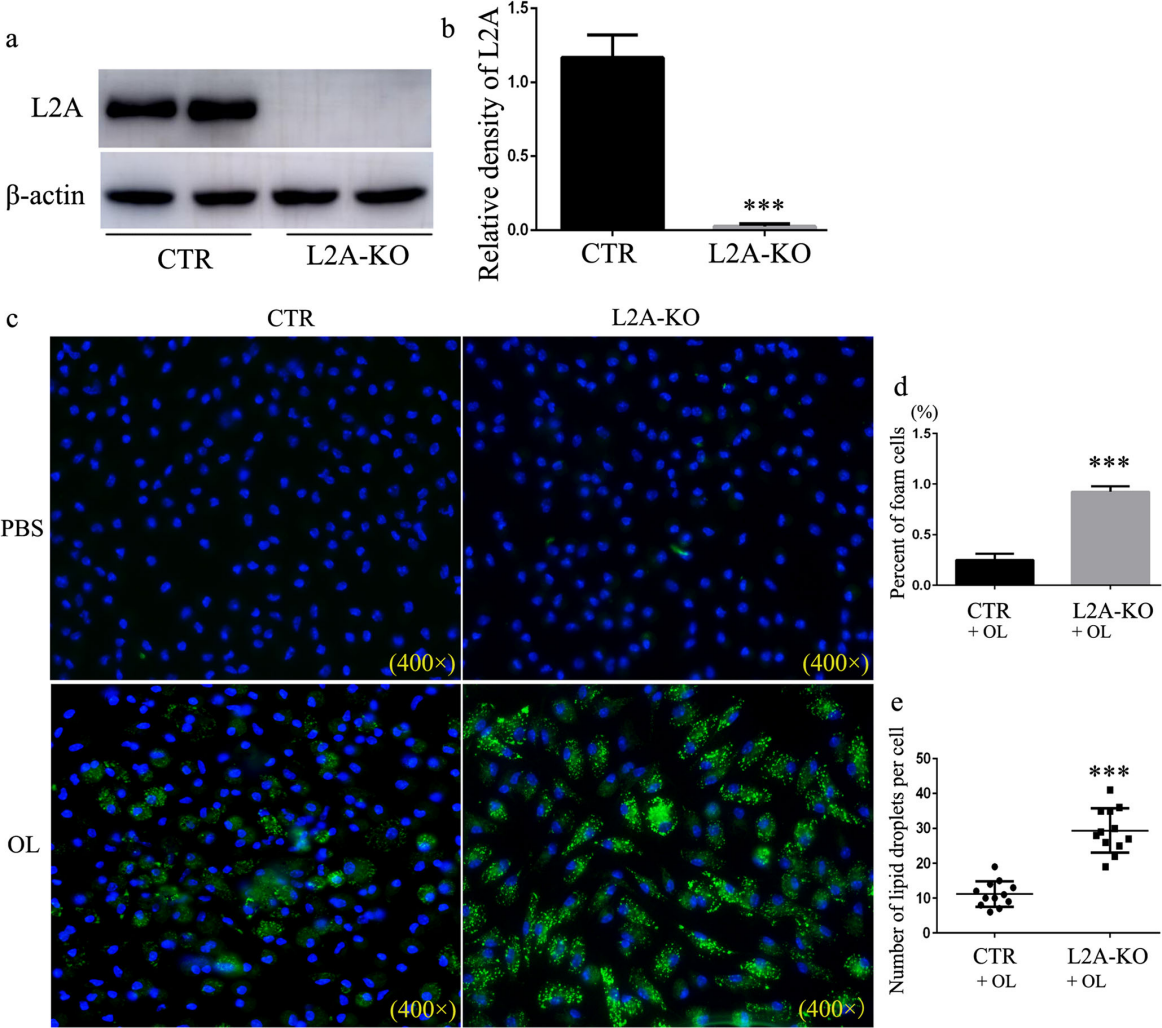
Analysis of the impact of CMA on lipid accumulation in macrophages. **a–b** Western blot for LAMP-2A (L2A) in macrophages isolated from macrophage-specific L2A-KO mice and control mice. **c** Macrophages were treated with OL (0.06 mM), and LDs were stained using Bodipy 493/503. **d** Percent of foam cells. **e** The number of LDs in each cells. ***p < 0.001 compared with control (CTR), *n* = 3

To study the consequences of defective CMA on macrophage lipid metabolism, we generated a macrophage-specific conditional knockout mouse for LAMP-2A to selectively block CMA in macrophage (Fig. 3a–b). Peritoneal macrophages isolated from L2A-KO mice transformed into more foam cells and internalized more lipids than that from control mouse visible with either BODIPY493/503 or oil-red O staining (Fig. 3c–e and Supplementary Fig. S1). These results suggest that CMA deficiency results in LD accumulation, indicating that CMA malfunction may be responsible for lipid accumulation during atherosclerosis progression.

### PLIN2 Degradation Is Not Involved in the Regulation of CMA on Lipid Metabolism

**Fig. 4:**
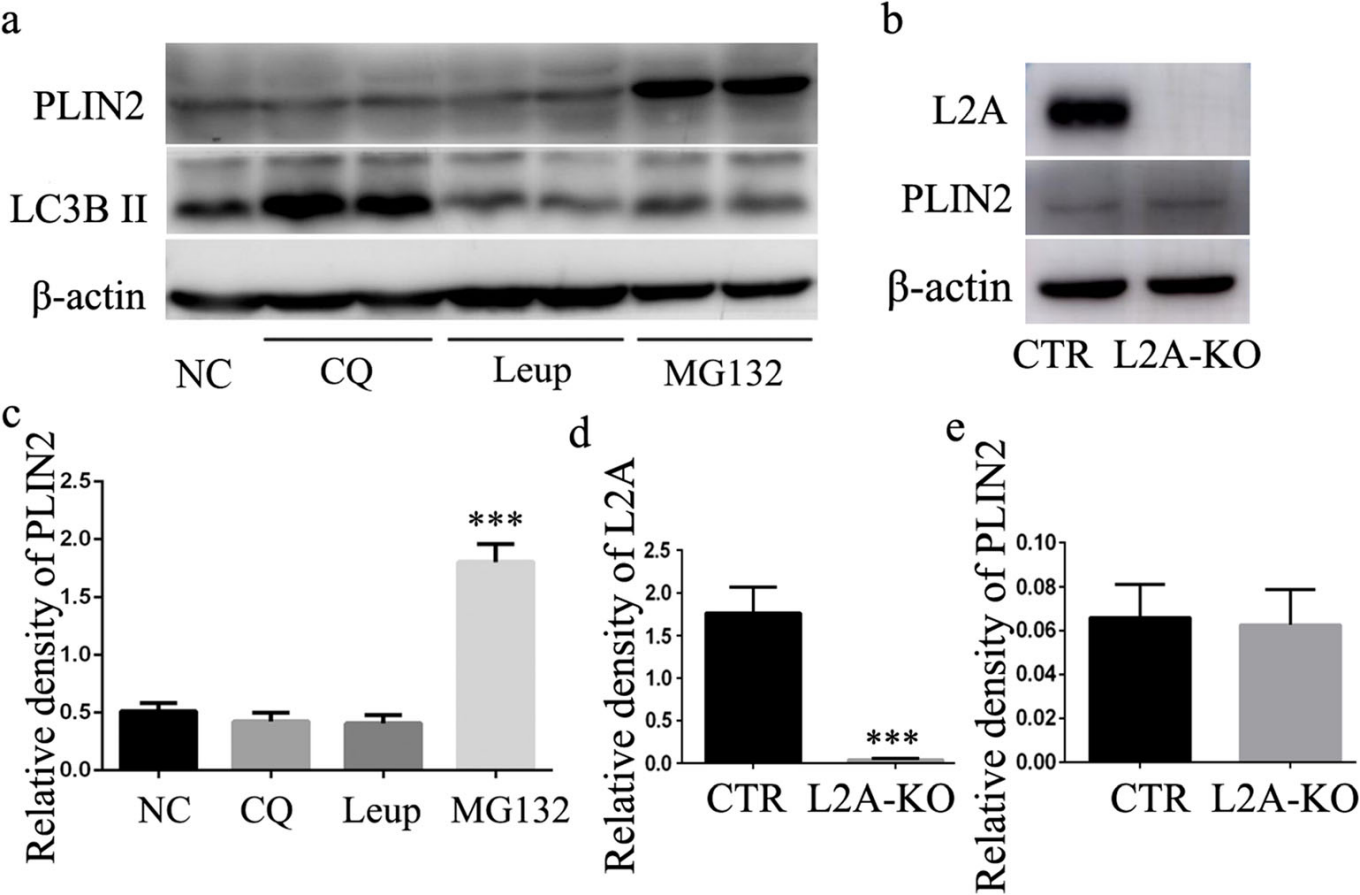
Investigation of the molecular mechanisms by which CMA regulates lipid accumulation in macrophage. **a** and **c** The effect of different protease inhibitors, MG132 (a proteasome inhibitor), leupeptin (trypsin-like and cysteine protease inhibitor), and CQ (a lysosomal protease inhibitor), on the degradation of PLIN2. **b**, **d,** and **e** Analysis of the impact of CMA deficiency on the degradation of PLIN2 by western blot. ***p < 0.001 compared with control (NC or CTR), *n* = 3

The pronounced accumulation of lipids observed in macrophages of L2A-KO mice (Fig. 3c and Supplementary Fig. S1) prompted us to explore possible alterations in lipid metabolism. As previous study showed that CMA blockage resulted in LD accumulation in hepatocytes because of dysfunctional lipolysis caused by inadequate degradation of lipid droplet-associated proteins (perilipin 2) [6]. To determine whether these findings in hepatocytes were recapitulated in macrophages, we first explored the contribution of the lysosome and proteasome system to the degradation of perilipin 2 (PLIN2) in macrophage. Macrophages were treated with MG132 (a proteasome inhibitor), leupeptin (trypsin-like and cysteine protease inhibitor), and CQ (a lysosomal protease inhibitor). As shown in Fig. 4a, addition of proteasome-specific inhibitor MG132 significantly increased PLIN2 level after treatment for 24 h. However, the other protease inhibitors had no effect on PLIN2 protein levels (Fig. 4 a and c). Note the increase of LC3-II level by CQ treatment validating inhibition of lysosomal degradation (Fig. 4a). These results indicated that lysosomal pathway was not involved in the degradation of PLIN2. Consistent with previous studies in fibroblastic cells [11], PLIN2 was more likely to be degraded through a proteasomal pathway in macrophage. Since lysosomes are the ultimate site for substrates degraded through CMA [5], these results suggest that PLIN2 may be not a CMA substrate in macrophage. To better illustrate the role of CMA in PLIN2 degradation, we analyzed PLIN2 level in macrophages from both control and L2A-KO mice and found no differences between the two groups (Fig. 4b–e). Together, we have to come to the conclusion that PLIN2 was not degraded through CMA in macrophage, and the increased lipid accumulation observed in CMA-deficient macrophage did not result from deficient PLIN2 degradation.

### CMA Modulates Macrophage Lipid Metabolism Through Lipid Regulatory Enzymes

**Fig. 5:**
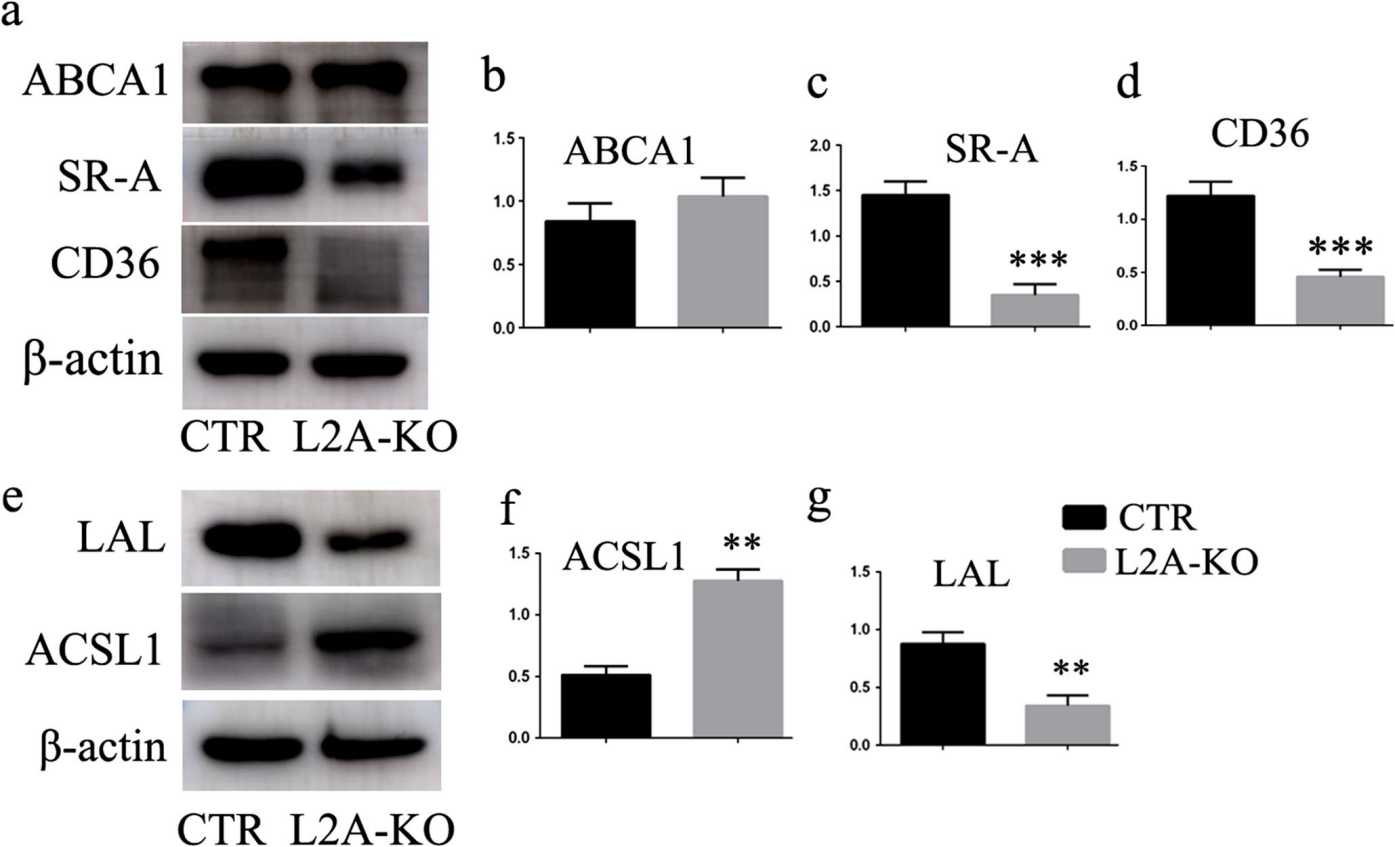
Investigation of the molecular mechanisms by which CMA regulates lipid accumulation in macrophage. **a**–**d** Analysis of the impact of CMA deficiency on proteins involved in lipid binding and transport, including SR-A, SR-B (CD36), and ABCA1. **e**–**g** Analysis of the impact of CMA deficiency on proteins involved in triglyceride synthesis (ACSL1) and lipid breakdown (LAL). **p < 0.01 and ***p < 0.001 compared with control (CTR), *n* = 3

We went on to explore possible alterations in lipid binding and transport, triglyceride synthesis, and cholesterol metabolism to search for the molecular mechanisms by which CMA affected lipid accumulation. We first studied the protein levels of SRs, including SR-A and SR-B (CD36), acting as receptors for membrane transport of modified low-density lipoprotein (LDL) and long-chain fatty acids [12, 13]. Surprisingly, both of the two SRs were reduced in macrophages of L2A-KO mice compared with that of CTR mice (Fig. 5a–d), indicating impaired ability of lipid uptake. Therefore, SRs could not be the factors that contributed to the marked lipid accumulation observed in L2A-deficient macrophage. Besides, ATP-binding cassette sub family A member 1 (ABCA1), a key gatekeeper influencing intracellular cholesterol efflux [14], was unchanged between the two groups (Fig. 4a–b). We next analyzed possible changes in lipid metabolism, including ACSL1 and LAL; lipid regulatory enzymes that catalyze synthesis of cellular lipids from long-chain fatty acids [15]; and the intracellular hydrolysis of cholesteryl esters and triglycerides [16], respectively. Encouragingly, ACSL1 was increased, and LAL was reduced in L2A-deficient macrophage (Fig. 5e–g), suggesting that CMA blockage caused enhanced lipid synthesis and impaired lipid breakdown in macrophage, which might underlie the basis of increased lipid accumulation in L2A-deficient macrophage.

## Discussion

CMA is a specialized form of autophagy, which is responsible for degradation of some proteins with a pentapeptide motif [5]. In the recent years, CMA is also identified as an important regulator of lipid catabolism through degradation of lipid droplet-associated proteins [6] and enzymes involved in lipid metabolism [7]. Deficient CMA caused marked lipid accumulation both in hepatocytes in vitro and in mouse liver [6, 7]. Herein, we reported that blockage of CMA in macrophage led to pronounced intracellular lipid accumulation. This is consistent with previous studies in hepatocytes, confirming a critical function for CMA in lipid metabolism.

Intracellular lipolysis is the process to hydrolyze triglycerides or cholesteryl esters to fatty acids (FAs). Apart from the classical “neutral lipolysis,” which degrades lipids with the help of cytoplasmic hydrolases, another pathway called “acid lipolysis” or “lipophagy” has been identified in the past several years [17–19]. Lipophagy refers to degradation of lipids through autophagy-lysosome pathway [19]. Lipophagy represents a hopeful discovery since it may be an efficient pathway to reduce cellular lipid load, which could have important implications for human diseases with lipid disorders such as atherosclerosis, fatty liver, and obesity [19, 20]. In this research, we found that lipophagy was impaired in advanced atherosclerotic lesions and its deficiency may be a cause of lipid accumulation and atherosclerosis progression. At present, it is still not clear how lipophagy is regulated in the organism. A recent study by Kaushik and Cuervo [5] provides compelling evidence for a new mechanism by which CMA affects lipophagy (acid lipolysis) and cytoplasmic neutral lipolysis in hepatocyte. This impressive research breaks people’s understanding of CMA: the only possible contribution of CMA to cellular metabolism was by providing free amino acids resulting from protein breakdown. It identified a new role of CMA in lipid and glucose metabolism and in overall organism energetics. Herein, we further verified this finding in macrophage. We also showed that excessive lipid accumulation in macrophage impaired both macroautophagy and CMA. These findings unveil a complex link between lipid metabolism and CMA and lead us to propose that atherosclerosis progression may be accompanied with CMA malfunction, which in turn accelerates lipid accumulation and atherosclerosis progression.

In the research by Kaushik and Cuervo in 2015 [5], they attributed the marked lipid accumulation in CMA-deficient cells to limited degradation of the lipid-associated proteins perilipin 2 and 3 (PLIN2 and PLIN3) by CMA, because they did detect PLIN2 in CMA-active lysosomes and its degradation in this compartment. However, as early as in 2005, PLIN2 (ADRP) has been reported to be degraded through the ubiquitin/proteasome pathway in fibroblastic cells [10], whereas other proteolytic processes, including lysosome pathway, were ineffective. This controversy prompted us to further explore whether the pronounced lipid accumulation in CMA-deficient macrophage was a consequence of limited degradation of PLIN2. Surprisingly, proteasome-specific inhibitor MG132 significantly increased PLIN2 level. But we did not find apparent changes in PLIN2 level in macrophage treated with CQ, a lysosomal protease inhibitor. This result is consistent with studies reported in 2005 [10] but inconsistent with recent studies published in 2015 [5]. We further analyzed PLIN2 level in both control and CMA-deficient macrophage and also found no differences. These results seem to suggest that degradation of PLIN2 through lysosomes or proteasomes is inscrutable and depends on the cellular requirement or the cell type. But it is certain that increased lipid accumulation in CMA-deficient macrophage is not associated with PLIN2 degradation.

But, what is the reason? The amount of lipid content in cell is directly related to how much it “eat” and “spit.” Surprisingly, SR-A and SR-B (CD36), acting as receptors for uptake of modified LDL and long-chain fatty acids, were reduced in CMA-deficient macrophage compared with control cells. ABCA1, a key gatekeeper influencing intracellular cholesterol efflux, was unchanged between the two groups. Therefore, lipids uptake or efflux was neither the factor that facilitated lipid accumulation upon CMA inhibition. In another study by Schneider JL et al. in 2014 [6], they reported that key enzymes in lipid metabolism were CMA substrates and impairment of their degradation through CMA contributed to the lipid accumulation observed in the liver of CMA-defective mouse. We therefore also analyzed possible changes in two enzymes involved in lipid metabolism, ACSL1 and LAL. ACSL catalyzes the acylation of fatty acids into long-chain acyl CoAs (LCA-CoAs), which is the first step in triglyceride synthesis and lipid storage after fatty acid entry into the cell [15]. Overexpression of ACSL1 increased lipid deposition in hepatic (HepG2) cells and rodent liver in vivo [15]. Correspondingly, LAL hydrolyzes cholesteryl esters and triglycerides in lysosomes to free cholesterol and free fatty acids [16]. Restoration of LAL by adenovirus-mediated gene transfer in LAL null (LAL(−/−)) mouse showed triglycerides and cholesterol reductions in the liver, spleen, and small intestine [21]. LAL is expected to be an attractive target to reduce lipid load in cells. Our results showed that ACSL1 was increased and LAL was reduced in L2A-deficient macrophage, suggesting that CMA blockage caused enhanced lipid synthesis and impaired lipid breakdown in macrophage, which may be responsible for the abnormal lipid accumulation in CMA-deficient macrophage. Reverse experiments of ACSL1 and LPL are needed to test the possibility in the future.

As macrophage foam cells are the most abundant cell populations in atherosclerotic plaque, understanding the role of CMA on lipid metabolism has important implications for atherosclerosis. Because there are no CMA inhibitors in the current, blockage of LAMP-2A by genetic intervention remains, to date, the most specific way to inhibit CMA. Therefore, it is vital to investigate the effect of CMA on atherosclerosis in animal study by crossing LAMP-2A-deficient mice with atherosclerosis-prone ApoE (−/−) or LDLR (−/−) mice. We look forward to more pharmacological studies targeting LAMP-2A to facilitate lipid metabolism and alleviate atherosclerosis progression in the future.

## Electronic Supplementary Material

ESM 1 (DOCX 1755 kb)

## Abbreviations

CMA: Chaperone-mediated autophagy
LAMP-2A: Lysosome-associated membrane glycoprotein 2, isoform A
OL: Oleate
PLIN2: Lipid-associated proteins perilipin 2
SR: Scavenger receptor
ABCA1: ATP-binding cassette sub family A member 1
ACSL1: Long-chain-fatty-acid-CoA ligase 1
LAL: Lysosomal acid lipase
HSC 70: Heat shock cognate 71 kDa protein
LDs: Lipid droplets
FAs: Fatty acids
